# Blurring the Boundaries: An Investigation of Eating Disorder Recovery Content on TikTok

**DOI:** 10.1002/eat.70153

**Published:** 2026-06-16

**Authors:** Scott Griffiths, Isabel Martyn, Harish Kannan, Ben Stone, Emma Austen, Edoardo Tescari, Usha Nattala, Wesley Grey, Noa Brittain, Deborah Mitchison, Jake Linardon, Jasmine Fardouly, Emily Harris

**Affiliations:** ^1^ Melbourne School of Psychological Sciences University of Melbourne Melbourne Australia; ^2^ Unforgettable.Me Melbourne Australia; ^3^ Melbourne Data Analytics Platform, School of Computing and Information Systems University of Melbourne Melbourne Australia; ^4^ Discipline of Psychology, Graduate School of Health University of Technology Sydney Sydney Australia; ^5^ School of Psychology Deakin University Melbourne Australia; ^6^ School of Psychology, Faculty of Science The University of Sydney Sydney Australia

**Keywords:** algorithms, eating disorders, pro‐eating disorder, recovery, social media, TikTok

## Abstract

**Objective:**

Eating disorder recovery content is widely circulated on TikTok. We thematically analyzed recovery content on TikTok, examined its associations with symptom severity among individuals with eating disorders, and assessed its co‐occurrence with pro‐eating disorder material within their TikTok feeds.

**Method:**

Study 1 was a qualitative reflexive thematic analysis of 250 TikTok posts containing eating disorder recovery hashtags. Study 2 was a quantitative examination of TikTok usage data and symptom severity collected from 42 individuals with eating disorders over a one‐month period.

**Results:**

Fifty percent of “recovery” posts contained pro‐eating disorder elements, including imagery or messaging consistent with disordered eating norms, weight comparisons, and thin‐ideal reinforcement. Quantitative analyses provided no evidence that greater exposure to recovery content was associated with lower or higher eating disorder symptom severity, although the study was underpowered to detect small‐to‐moderate effects. Recovery and pro‐eating disorder content were strongly correlated within participants' TikTok feeds in terms of the volume of videos delivered (Spearman's *ρ* = 0.91) and their proportional representation (*ρ* = 0.77), with the former association remaining high even after controlling for overall TikTok exposure (*ρ* = 0.81).

**Discussion:**

Our findings raise concerns about the safety of TikTok recovery spaces. TikTok content pitched as “eating disorder recovery” frequently contains pro‐eating disorder features, shows no strong protective association with symptom severity, and is regularly encountered alongside pro‐eating disorder content within users' feeds. We conclude that overlap between recovery content and pro‐eating disorder content operates at two levels: within individual videos and across the broader feed context.

## Summary

1. Half of TikTok posts tagged as eating disorder recovery contained pro‐eating disorder elements, including thin‐ideal reinforcement, weight comparisons, and imagery consistent with disordered eating norms, suggesting blurred boundaries between recovery‐oriented and harmful content.
2. Among individuals with eating disorders, exposure to recovery content was not associated with lower symptom severity over 1 month.
3. Recovery and pro‐eating disorder content were highly correlated within users' feeds, both in absolute volume and proportional representation, indicating that recovery content occurs alongside broader pro‐eating disorder content.

## 1 Introduction

Social media platforms are a popular source of information for people recovering from eating disorders. TikTok is particularly relevant in this context because its algorithmically curated “For You” feed prioritizes content discovery based on engagement signals, meaning users are frequently exposed to content beyond the accounts they explicitly follow (Griffiths et al. 2024). Recovery from an eating disorder is fragile and non‐linear, with high relapse rates (Miskovic‐Wheatley et al. 2023), and social media messaging may either reinforce or destabilize recovery‐related cognitions and behaviors. Although pro‐eating disorder social media content is well documented and associated with worse disordered eating outcomes (Arseniev‐Koehler et al. 2016; Custers 2015; Goh et al. 2022; Jett et al. 2010; Rodgers et al. 2015), less is known about the validity of social media content labeled as eating disorder recovery, which may be contaminated with pro‐eating disorder rhetoric (e.g., Herrick et al. 2021; Greene et al. 2023). We also know little about whether exposure to eating disorder recovery content is associated with positive outcomes among individuals with eating disorders, and to what extent it co‐occurs with pro‐eating disorder content in the feeds of social media users. Our study aimed to examine (i) the validity of recovery‐labeled content on TikTok, (ii) whether exposure to such content was associated with eating disorder symptom severity, and (iii) the extent to which recovery and pro‐eating disorder content co‐occurred within algorithmically curated social media feeds.

### 1.1 Social Media Content About Eating Disorders

#### 1.1.1 Eating Disorder Recovery Content

Our working definition of “eating disorder recovery” was adopted from Bardone‐Cone et al. (2010): namely, that eating disorder recovery entails (i) restoration of physical health, (ii) cessation of disordered eating behaviors, and (iii) restoration of psychological health. Eating disorder recovery content (referred to as “recovery content” hereafter) refers to social media content that purportedly aims to support people recovering from an eating disorder. On TikTok, such content is typically organized around hashtags such as #EDrecovery and diagnosis‐specific variants (e.g., #ANrecovery).

Three qualitative studies suggest that recovery‐labeled TikTok content is not uniformly supportive. Herrick et al. (2021) analyzed 150 posts tagged #EDrecovery and identified themes including eating disorder awareness, inpatient narratives, eating in recovery, transformations, and “trendy gallows humour.” The latter two raised concerns: “transformation” videos frequently adopted weight‐loss aesthetics resembling thinspiration before reframing toward recovery, while humor‐based content often normalized or trivialized symptoms using imagery such as rice cakes, gum, calorie‐tracking screenshots, and BMI calculators. For example, some videos presented stereotypical “ED meals” consisting only of gum or rice cakes as humorous or relatable content or began with emaciated body‐check images before transitioning to captions about recovery. Greene et al. (2023) analyzed 241 recovery posts and found that diet culture appeared in 44.4% of videos, including weight‐loss framing of recovery, restrictive dietary rules, and calorie quantification, with weight‐loss framing and calorie counting particularly common in binge eating disorder recovery content. More recently, Bloxham et al. (2025) analyzed 312 recovery‐tagged TikTok videos and similarly reported blurred boundaries between recovery discourse and symptom‐reinforcing content. Collectively, these qualitative studies indicate that recovery‐labeled content on TikTok may reproduce elements associated with pro‐eating disorder norms and are consistent with research on other social media platforms, such as Instagram (e.g., Goh et al. 2022). To date, however, no quantitative research has examined whether exposure to such content is associated with symptom severity. Examining whether exposure to such content is associated with eating disorder symptom severity is therefore important, given the possibility that recovery‐labeled content may simultaneously support and undermine recovery processes.

#### 1.1.2 Pro‐Eating Disorder Content

Pro‐eating disorder content refers to social media content that primarily and explicitly aims to reinforce disordered eating norms (Rodgers et al. 2015). Pro‐eating disorder content has its origins in “pro‐ana” (pro‐anorexia) online communities that framed anorexia nervosa as a lifestyle rather than an illness and provided thinspiration, calorie restriction strategies, and validation for behaviors that are targeted as clinical symptoms in treatment settings (Boero and Pascoe 2012; Norris et al. 2006). Contemporary pro‐eating disorder content has evolved into more diffuse and platform‐adapted forms that circulate across mainstream social media environments. This material includes instructional content for developing, maintaining, or concealing an eating disorder; the normalization of restrictive, binge‐eating, or purging behaviors; and “thinspiration” or “fitspiration” imagery designed to motivate users toward extreme thinness or fitness (e.g., Arseniev‐Koehler et al. 2016; Custers 2015; Goh et al. 2022; Peebles et al. 2012).

Pro‐eating disorder messaging ranges from overt advocacy to subtler reinforcement of disordered norms. Some content is explicitly harmful, including diet culture rhetoric and fat‐shaming. Other triggers are more indirect, including caloric disclosure, extreme body‐shaping routines, and comparison‐oriented trends that foreground weight, shape, and control (Arthur‐Cameselle et al. 2018; Kenny and Lewis 2023). Exposure to such material is associated with greater disordered eating behaviors (e.g., Jett et al. 2010; Rodgers et al. 2015) and overall symptom severity (Griffiths and Stefanovski 2019; Griffiths et al. 2024).

### 1.2 Social Media Algorithms and Eating Disorder Content

Algorithmic recommendation systems curate and prioritize social media content based on predictions about what will maximize user engagement (Griffiths et al. 2024). Modern social media platforms no longer present content in reverse chronological order (e.g., Facebook circa 2008) but instead use machine learning models that analyze users' past behaviors, interactions, and content preferences to select and sequence posts for each user's feed (Lasser and Poechhacker 2025; Griffiths et al. 2024). These systems are optimized to increase engagement metrics such as watch time, session length, and clicks, which can inadvertently promote content that is engaging but potentially harmful. Recommendation systems may suggest both eating disorder recovery content and pro‐eating disorder content to users with an eating disorder because both forms of content are more likely to resonate with their experiences and therefore generate engagement signals that guide future recommendations (Golbeck 2024; Griffiths et al. 2024; Lasser and Poechhacker 2025). We refer to this connection between distinct content themes that nonetheless both engage the user as “algorithmic linkage.” Evidence for algorithmic linkage between recovery and pro‐eating disorder content comes from work on social media platforms other than TikTok. Golbeck (2024) examined recommendation patterns on Pinterest following interaction with eating disorder recovery content. They used a combination of content analysis and platform queries to show that when users viewed recovery‐related posts, the system subsequently recommended eating disorder–reinforcing material, indicating that recovery and pro‐eating disorder content can be algorithmically adjacent. This finding suggests a mechanism by which recovery seekers might be exposed to harmful material through existing recommendation pathways.

Qualitative research suggests that users experience patterns of exposure consistent with possible algorithmic linkage between recovery and pro‐eating disorder content, and that these patterns can be difficult to manage. Parnell et al. (2026) interviewed 17 young adults with lived experience of eating disorders in the United Kingdom and identified themes including “View One, See More” and “From Helpful to Unhelpful,” describing the experience of watching a single algorithm‐delivered recovery video on TikTok and then being delivered a cascade of harmful videos, including pro‐eating disorder videos. Similarly, Praet et al. (2024) interviewed seven adolescent girls with anorexia nervosa, who reported that eating disorder material was difficult to avoid on social media in general. We speculate that even when users deliberately seek recovery‐oriented material, recommendation systems may continue to surface adjacent pro‐eating disorder content if engagement patterns predict sustained attention, and this may particularly be the case if the user has engaged with this material in the past.

#### 1.2.1 TikTok

TikTok provides a particularly relevant platform for examining the algorithmic dynamics of recovery and pro‐eating disorder content. Its “For You Page” relies on multiple machine learning models that heavily weight user behavior to personalize content exposure (Griffiths et al. 2024). With more than 1.8 billion users worldwide, predominantly adolescents and young adults (Boeker and Urman 2022), the platform reaches the demographic group most vulnerable to eating disorders (Galmiche et al. 2019).

### 1.3 Study Aims and Hypotheses

Our study aimed to examine the validity, psychological correlates, and algorithmic context of eating disorder recovery content on TikTok. First, we qualitatively analyzed posts tagged with recovery‐related hashtags to identify and categorize the presence and structure of pro‐eating disorder contamination within recovery‐labeled content. Second, we quantitatively examined whether exposure to, and engagement with, recovery‐labeled posts were associated with symptom severity among individuals with eating disorders. Third, we assessed the extent to which recovery and pro‐eating disorder posts co‐occurred within users' algorithmically curated feeds. We hypothesized that (i) recovery‐labeled content would be contaminated by pro‐eating disorder elements, and (ii) recovery and pro‐eating disorder posts would demonstrate significant algorithmic co‐occurrence (i.e., be positively correlated within participants). We did not form an a priori hypothesis about whether exposure to recovery content would be associated with symptom severity because existing research (Herrick et al. 2021; Greene et al. 2023; Bloxham et al. 2025) and our own intuition suggested that this material could be both beneficial and harmful, depending on the extent of contamination with pro‐eating disorder content.

## 2 Method

### 2.1 Qualitative Study

#### 2.1.1 Identification and Retrieval of Eating Disorder Recovery Videos

We compiled a list of 37 eating disorder recovery‐related hashtags through manual searches of TikTok, consultation with clinicians specializing in eating disorders, and review of relevant literature (Griffiths et al. 2024; Herrick et al. 2021; Sukunesan et al. 2021; Zhou et al. 2020). Hashtags were ranked by total post volume, and the ten most frequently used were selected for analysis: #recoveryispossible, #recoverytok, #EDRecovery, #foodfreedom, #edsheeranrecoveryy, #EDawareness, #arfidrecovery, #anarecovery, #bedrecovery, #miarecovery. Two anorexia‐specific hashtags (#Anarec0very and #anarecovery) initially ranked in the top ten but were replaced to enhance diagnostic breadth, as anorexia nervosa was already represented. They were substituted with the next most popular recovery hashtags corresponding to other diagnoses (#bedrecovery and #miarecovery). These ten hashtags represent 2.18 million videos or 27% of all identified recovery hashtags in our sampling frame and represent double the number of hashtags examined in prior hashtag‐based qualitative studies of TikTok recovery communities, which have analyzed either a single hashtag (e.g., #EDrecovery; Herrick et al. 2021) or five hashtags (Bloxham et al. 2025; Greene et al. 2023).

For each of the ten selected hashtags, the first 25 posts returned under TikTok's default “Top” search ordering were collected on 22 June 2025, yielding an initial sample of 250 posts. This cut‐off balanced breadth and depth and is similar in size to published hashtag‐based qualitative analyses of TikTok recovery content (Bloxham et al. 2025; Greene et al. 2023; Herrick et al. 2021). URLs were extracted using an incognito browser without logging into a TikTok account to reduce account‐level algorithmic personalization, although device‐ and location‐level curation, as well as curation based on language settings (i.e., English), likely persisted. Duplicate posts appearing under multiple hashtags were identified and removed, and then replaced by the next unique post. For each post, audio, captions, on‐screen text, and salient visual elements (e.g., body‐focused imagery, food imagery, text overlays, and environmental context) were systematically recorded in an Excel spreadsheet. All posts were viewed at least twice by the lead qualitative researcher (second author) to ensure accuracy and completeness of data extraction.

#### 2.1.2 Thematic Analysis

Braun and Clarke's (2006) six‐phase reflexive thematic analysis was used to generate themes from the data, involving iterative familiarization, systematic coding, and the development and refinement of themes prior to producing the analytic narrative. A predominantly inductive approach was used, with some deductive sensitivity retained to ensure analytic relevance to prior literature (e.g., literature describing pro‐eating disorder norms, thinspiration aesthetics, and diet culture elements). Codes were developed at both semantic and latent levels, capturing content communicated explicitly through the videos, as well as underlying assumptions and meanings. Analysis was iterative and recursive, meaning that movement between data, codes, and developing themes occurred throughout. Following initial coding, candidate themes were constructed by grouping conceptually related codes. Consistent with Braun and Clarke's (2021) conceptualization of reflexive thematic analysis, themes were treated as interpretive analytic outputs developed through researcher engagement with the data, rather than as objectively emergent categories. These themes were reviewed against both coded extracts and the full dataset, refined to ensure internal coherence and clear distinction between themes, and defined around central organizing concepts. Themes were developed to represent patterned meaning relevant to the research question, rather than to quantify topic prevalence. Coding and theme development were led by the second author, with regular analytic discussions with the first author to challenge interpretations and sharpen theme boundaries. Regarding positionality, the second author acknowledges that she does not have lived experience of an eating disorder but does have experience supporting others with eating disorders. The first author also does not have lived experience of an eating disorder but has extensive research and clinical experience working with people with eating disorders.

### 2.2 Quantitative Study

#### 2.2.1 Participants

Participants were 42 individuals with active, clinically diagnosed eating disorders. The sample comprised 39 females and 3 participants who did not disclose their gender, aged 16 to 38 years (*M* = 21.88, SD = 5.04). The majority (76.2%) had a diagnosis of anorexia nervosa, followed by bulimia nervosa (7.1%), binge eating disorder (4.8%), and OSFED or UFED (4.8%). Participants were recruited through advertisements distributed to psychology undergraduates at the University of Melbourne and via Australian eating disorder support organizations. Inclusion criteria wereas follows: (i) aged 16 years or older, (ii) TikTok use within the previous month, (iii) English set as the primary language setting on TikTok, and (iv) fluency in English. The sole exclusion criterion was submission of a TikTok data file containing fewer than 50 posts, a threshold implemented to reduce the likelihood of fraudulent or invalid responses—one participant was excluded on this basis.

#### 2.2.2 Procedure

Participants requested their TikTok user data directly through the TikTok application, which provides downloadable data files in JSON format. Each file contained unique URLs corresponding to videos delivered to the participant, and these URLs were used to scrape metadata for posts consumed during the preceding month. Scraping was conducted using an automated script with dynamic IP rotation and human‐like request timing to minimize detection by TikTok's anti‐scraping systems. Retrieved metadata included post hashtags and engagement indicators (e.g., whether the participant “liked” the post). Metadata were merged with participants' self‐report survey data, and posts were classified into content categories based on hashtags. For each participant, the number and proportion of posts delivered and liked within each category were calculated.

#### 2.2.3 Identification of Recovery and Pro‐Eating Disorder Videos

Posts were classified based on the presence of recovery‐ or pro‐eating disorder‐related hashtags. Initial seed hashtags for each category were derived from prior literature (Herrick et al. 2021; Greene et al. 2023; Sukunesan et al. 2021; Zhou et al. 2020; Griffiths et al. 2024) and refined through TikTok exploration and domain expertise. Example recovery hashtags included #edrecovery and #anarecovery, whereas pro‐eating disorder hashtags included #thinspo, #thinspire, and #proana.

To extend coverage beyond a fixed predefined list, we implemented fuzzy string matching using the RapidFuzz library (Bachmann 2022), which applies Levenshtein‐distance‐based similarity scoring to detect orthographically similar strings. Similarity thresholds of 90% and 95% were evaluated; a threshold of 95% was ultimately selected as it provided an optimal balance between sensitivity and precision. This approach enabled systematic detection of closely related and intentionally misspelled variants (e.g., #proana → #proaana; #edrecovery → #edrec0very), which are frequently used to evade moderation filters. Hashtags identified via RapidFuzz were manually reviewed to remove false positives and confirm semantic equivalence prior to inclusion. Compared to approaches relying solely on static, predefined hashtag lists (e.g., Griffiths et al. 2024), this method improves detection of evolving and intentionally altered hashtag forms. More precise quantification of recovery and pro‐eating disorder content reduced measurement error and improved our ability to detect true associations.

Approximately 25% of our identified videos contained both recovery and pro‐eating disorder hashtags and were coded as a separate “combination” category. We did not include this variable in our primary analyses because it represents a mixed exposure that overlaps conceptually with both recovery and pro‐eating disorder content, reducing interpretability. In the interest of being comprehensive, and for quality control, we conducted sensitivity analyses including these videos, but they did not change the pattern of results. Accordingly, the more parsimonious models are presented here.

#### 2.2.4 Diagnosis Status, Eating Disorder Symptoms, and Demographics

Participants completed self‐report measures including current eating disorder diagnostic status, eating disorder symptom severity, and demographics. Diagnostic status was assessed with the item, “Are you currently diagnosed with an eating disorder?” (yes/no), accompanied by clarification that “diagnosed” referred to formal confirmation by a qualified health professional (e.g., psychologist, psychiatrist, or medical practitioner). Eating disorder symptoms were measured using the Eating Pathology Symptom Inventory (EPSI; Forbush et al. 2013), which assesses symptom severity over the past month. Internal consistency in the present sample was good (Cronbach's *α* = 0.85; McDonald's *ω* = 0.81), consistent with reliability estimates reported in the original validation study (*α* = 0.84–0.89; Forbush et al. 2013).

#### 2.2.5 Data Analyses

Analyses were conducted to examine (i) the relationship between recovery and pro‐eating disorder content within participants' TikTok feeds and (ii) whether exposure to recovery content was associated with eating disorder symptom severity. Exposure was operationalized using two metrics: the number of posts delivered (count variables) and the proportion of all delivered posts (proportion variables). Exposure to recovery content was operationalized as both the total number of recovery videos delivered to a user and the proportion of recovery videos relative to all videos delivered. Because count variables were positively skewed, associations among exposure variables were examined using Spearman's rank‐order correlations. Multiple linear regression models were then used to evaluate associations between content exposure and eating disorder symptom severity. Two models were specified to avoid structural dependency between count and proportion metrics. The first model included count‐based exposure variables (number of pro‐eating disorder posts delivered and number of recovery posts delivered). The second model included proportion‐based exposure variables (proportion of delivered posts that were pro‐eating disorder and proportion that were recovery‐related). Collinearity diagnostics indicated acceptable tolerances (> 0.30) and variance inflation factors (< 3.39; Alin 2010).

Given our modest‐sized clinical sample (*N* = 42), power analyses were conducted to determine the minimum effect sizes detectable with 80% power (*α* = 0.05, two‐tailed). For Spearman's rank‐order correlation, we achieved a conventional amount of power (80%) to detect associations of |*ρ*| = 0.41. In summary, we had adequate power to detect moderate‐to‐large correlations, were under‐powered for moderate associations (|*ρ*| ≈ 0.30, power = 50%), and were severely under‐powered to detect small (|*ρ*| ≈ 0.10, power = 9%) associations. Similarly, for our multiple linear regressions, we were adequately powered to detect moderate‐to‐large associations, with decreasing power to detect moderate, and especially small, effects. These power constraints reflect the practical challenges in conducting correlational research with clinically diagnosed samples, where recruitment is more difficult compared to non‐clinical populations.

### 2.3 Ethics Statement

This study was approved by the Human Research Ethics Committee at the University of Melbourne (ethics IDs: 23072 and 32,634). All participants provided informed consent prior to participation.

## 3 Results

### 3.1 Qualitative Results—Thematic Analysis of Recovery‐Labeled Content on TikTok

Using reflexive thematic analysis, we identified three overarching content patterns within eating disorder recovery‐tagged TikTok posts: (1) recovery‐oriented themes, (2) disorder‐reinforcing themes, and (3) ambivalent or mixed‐signal themes. Each of these themes had three subthemes. Themes and sub‐themes were not mutually exclusive; individual posts frequently contained features from more than one theme. In total, recovery‐oriented themes appeared in 58.4% of the sampled posts, disorder‐reinforcing themes in 50.0%, and ambivalent or mixed‐signal themes in 27.6%.

#### 3.1.1 Recovery‐Oriented Themes

Recovery‐oriented themes reflected content that was clearly aligned with recovery principles and explicitly positioned eating disorder thoughts and behaviors as targets of change. The sub‐theme *Encouraging Recovery Through Challenging the Eating Disorder* captured posts in which creators directly confronted disordered cognitions and modeled cognitive or behavioral shifts; for example, one user challenged guilt caused by her disorder, describing “When I came home after the hospital that helped me so much! If doctors were there telling me that it was ok to eat that amount to heal then why would I feel guilty about an extra cookie today?” Demonstrating active reframing of guilt and permission to eat. The sub‐theme *Educating and Raising Awareness About Eating Disorders* involved creators adopting an instructional role and correcting misconceptions about diagnosis, appearance, and legitimacy, such as stating, “You can't always tell if someone has an ed or not. Because an ed is a mental disorder, not a weight disorder,” thereby challenging weight‐based stereotypes and increasing mental health literacy. The sub‐theme *Authentic and Encouraging Narratives of Recovery* encompassed personal accounts that acknowledged recovery as emotionally difficult and non‐linear, and acknowledged setbacks while still framing it as worthwhile, often accompanied by affirmational language such as “FOOD IS NOT AN ENEMY, FUN, MEMORIES, RELATIONSHIPS, FUEL, ADVENTURE,” positioning recovery as both demanding and meaningful.

#### 3.1.2 Disorder‐Reinforcing Themes

Disorder‐reinforcing themes reflected content that validated, normalized, or subtly promoted eating disorder‐related beliefs and behaviors. The sub‐theme *Encouraging Pro‐eating Disorder Rhetoric and Behaviors* captured posts that explicitly or implicitly endorsed harmful practices, including references to the “ed to gym pipeline!” Alongside footage of illness and gym routines, suggesting symptom substitution or the continuation of compulsive patterns under the guise of self‐improvement. The sub‐theme *Romanticization and Normalization of Eating Disorders* involved aestheticized, emotionally stylized, or “edgy” portrayals of starvation and suffering, such as describing starvation as producing euphoria and akin to addiction: “…and starving yourself can make you feel euphoric, like a drug addict, or an alcoholic. What you crave is the numbing of the thing you don't wanna feel.” The sub‐theme *Quantifying Bodies and Food* included posts foregrounding measurement, comparison, and numerical tracking, including “before and after” transformations and “what I eat in a day” videos displaying portion sizes or calorie counts, reinforcing preoccupation with weight, intake, and bodily surveillance.

#### 3.1.3 Ambivalent and Mixed‐Signal Themes

Ambivalent and mixed‐signal themes included posts that did not clearly promote recovery or reinforce disorder, but instead contained elements of both. The sub‐theme *Use of Eating Disorders for Promotional Content* involved creators embedding products or services within recovery narratives, for example, “I was literally always cold… This app helped me recover,” blurring recovery messaging with commercial interests and potentially positioning these products and services as central to recovery. The sub‐theme *Gritty and Honest Realities of Eating Disorders and Recovery* captured confronting depictions of lived experience, including distressing thoughts or difficult treatment encounters presented without consistent reframing; for example, one post recreated a user's interpretation of a therapy interaction: “Hi fat pig… proceed to lock yourself in your room, far away from the kitchen. Oink oink.” These posts could validate lived experience and reduce isolation, but in some cases may also discourage help‐seeking or reinforce negative perceptions of treatment. The sub‐theme *Social Support and Collective Recovery* centered on shared identification and communal disclosure, and often used inclusive language to foster community among viewers. Creators often posed questions to audiences, such as: “Tell me your most unhinged thing you did because of food noise.” While such content could reduce shame and foster connection, it could also consolidate identification around shared illness experiences and normalize disordered norms, reflecting its mixed nature.

### 3.2 Quantitative Results—Exposure, Symptoms, and Algorithmic Co‐Occurrence

#### 3.2.1 Descriptive Statistics and Correlations

As shown in Table 1, participants received a mean of 5871 TikTok videos over the one‐month period, of which 139 (3.3%) were recovery‐related and 39 (1.2%) were pro‐eating disorder videos. Neither the number nor proportion of recovery or pro‐eating disorder videos delivered was significantly associated with eating disorder symptom severity. Greater overall TikTok exposure was moderately associated with receiving more recovery and pro‐eating disorder videos. In contrast, recovery and pro‐eating disorder exposure were strongly positively associated with one another, both in terms of the number of videos delivered (*ρ* = 0.91, *p* < 0.001) and their proportional representation within feeds (*ρ* = 0.77, *p* < 0.001), indicating that participants who received more recovery‐oriented content also tended to receive more pro‐eating disorder content. Importantly, the former association remained high when controlling for the overall number of TikTok videos delivered (partial *ρ* = 0.81, *p* < 0.001), indicating that the co‐occurrence of recovery and pro‐eating disorder content was not simply attributable to heavier overall TikTok use.

**TABLE 1:** Descriptive statistics and Spearman correlation coefficients for key quantitative variables.

Variable	*M*	Mdn	SD	Range	Spearman's correlation coefficient (*ρ*)					
					1	2	3	4	5	6
1. Total number of TikTok videos delivered	5871.44	4201.50	5109.33	62–16,911	—	0.54*	−0.05	0.59*	0.23	0.12
2. Total number of recovery videos delivered	139.36	67	179.65	0–750		—	0.71*	0.91*	0.80*	−0.08
3. Proportion that were recovery videos	0.033	0.020	0.042	0–0.158			—	0.59*	0.77*	−0.08
4. Total number of pro‐ED videos delivered	39.10	13.50	49.91	0–157				—	0.87*	−0.01
5. Proportion that were pro‐ED videos	0.012	0.003	0.032	0–0.202					—	0.08
6. Eating disorder symptoms	2.77	2.80	0.55	1.69–4.02						—

*Note:* Data for variables representing proportions are given to 3 decimal places instead of 2.

Abbreviation: Pro‐ED, pro‐eating disorder.

* *p* < 0.001.

#### 3.2.2 Multiple Regressions

The first linear regression model examined whether the number of recovery‐related and pro‐eating disorder videos delivered were associated with eating disorder symptom severity. As shown in Table 2, the overall model was not statistically significant. Neither recovery video exposure nor pro‐eating disorder video exposure was significantly associated with symptom severity. Also shown in Table 2, the second linear regression model examined whether the proportion of recovery‐related and pro‐eating disorder videos delivered were associated with symptom severity. The overall model was not statistically significant. Neither the proportion of pro‐eating disorder videos nor the proportion of recovery videos was significantly associated with symptom severity. Collinearity diagnostics again indicated acceptable tolerances (> 0.57) and variance inflation factors (1.76).

**TABLE 2:** Summary statistics from the multiple regressions.

Model	*F* (2, 39)	*R* ^2^	*p*	Predictor	*b*	SE	*β*	*p*
1	0.93	0.05	0.404	Number of recovery videos	−0.001	0.001	−0.34	0.244
				Number of pro‐eating disorder videos	0.002	0.003	0.18	0.536
2	1.06	0.05	0.357	Proportion of recovery videos	−2.97	2.70	−0.23	0.277
				Proportion of pro‐eating disorder videos	0.02	3.52	0.01	0.995

## 4 Discussion

Our study found that eating disorder recovery content on TikTok (i) is frequently contaminated with disorder‐reinforcing elements, (ii) shows no detectable protective association with symptom severity (with the caveat that we had low statistical power to detect small‐to‐medium associations), and (iii) is structurally intertwined with pro‐eating disorder material within personalized feeds.

### 4.1 Validity of Recovery‐Labeled Content on TikTok

Our qualitative analysis revealed substantial contamination of recovery‐labeled TikTok content with pro‐eating disorder elements. Half of all sampled posts contained disorder‐reinforcing features, indicating that recovery hashtags do not reliably demarcate recovery‐aligned material. Disorder‐reinforcing themes included explicit or implicit validation of disordered cognitions and behaviors, romanticized portrayals of starvation or illness, and quantification of bodies and food. These findings align with prior qualitative research documenting blurred boundaries within recovery hashtags on TikTok (Greene et al. 2023; Herrick et al. 2021; Parnell et al. 2026). Importantly, this estimate is conservative. If ambivalent and mixed‐signal themes were also considered forms of contamination, the proportion of recovery‐labeled content containing potentially disorder‐reinforcing elements would be considerably higher. For example, posts depicting distressing treatment encounters without clear recovery framing, prompts inviting users to share extreme behaviors linked to “food noise,” or recovery narratives embedded within promotional content may reduce shame or validate experience, but can also reinforce illness‐based norms or undermine help‐seeking. Taken together, recovery‐labeled TikTok spaces appear to contain a substantial amount of content that is not clearly recovery‐supportive.

### 4.2 Associations Between Recovery Exposure and Symptom Severity

The second aim examined whether exposure to recovery‐labeled content was associated with eating disorder symptom severity among individuals with clinically diagnosed eating disorders. Greater exposure to recovery‐labeled content was not significantly associated with either lower or higher symptom severity, and this finding was not contingent on our decision to exclude the substantial minority of videos (25%) that contained both recovery and pro‐eating disorder hashtags. However, these null findings should be interpreted cautiously. Power analyses indicated that the study was adequately powered to detect moderate‐to‐large associations but underpowered to detect small‐to‐moderate associations, which are more likely to characterize any true effect given the multifactorial nature of eating disorder risk and severity. What we can conclude, however, is that within the limits of this modest clinical sample we detected no strongly protective or harmful association between exposure to recovery‐labeled TikTok content and symptom severity. We also note that the thematic overlap between recovery content and pro‐eating disorder content observed in our qualitative study was mirrored in our quantitative study, insofar as 25% of all videos containing recovery hashtags were also found to contain at least one pro‐eating disorder hashtag. One possible explanation is that content creators and/or content sharers deliberately use overlapping terminology and hashtags to increase the visibility of their content within both recovery and pro‐eating disorder TikTok communities.

### 4.3 Algorithmic Co‐Occurrence of Recovery and Pro‐Eating Disorder Content

The third aim examined whether recovery and pro‐eating disorder content co‐occurred within participants' algorithmically curated feeds. Here, the findings were striking: recovery and pro‐eating disorder exposure were strongly positively correlated, both in terms of the number of posts delivered and their proportional representation within feeds. Participants who received more recovery‐oriented content also received more pro‐eating disorder content within the 1‐month observation window. Importantly, this association remained high when controlling for overall TikTok exposure, suggesting the co‐occurrence of recovery and pro‐eating disorder content was not simply due to heavier overall TikTok use. This pattern suggests a structural coupling of recovery and disorder‐reinforcing material within personalized TikTok feeds. By structural coupling, we refer to the erosion of separation between recovery and pro‐eating disorder content within the same algorithmically curated feed environment, rather than distinct hashtag communities. Our findings do not establish that TikTok's recommendation system intentionally promotes harmful material, nor do they permit causal inference regarding algorithmic mechanisms. However, they indicate that, in practice, recovery content is typically encountered within feed environments that also contain substantial pro‐eating disorder material. Taken together with the qualitative findings, contamination appears to operate at two levels: within individual posts and across the broader feed context. Even if a given recovery post is recovery‐aligned, it is likely embedded within a content ecosystem that includes and preferentially surfaces disorder‐reinforcing material.

### 4.4 Clinical and Practical Implications

The clinical implications of our findings warrant consideration. Although we did not detect a worsening association between recovery exposure and symptom severity, we also found no clear evidence of a protective association in this sample. Concurrently, half of recovery‐labeled posts contained disorder‐reinforcing elements, and recovery exposure was strongly coupled with exposure to pro‐eating disorder material within feeds. Given prior research linking pro‐eating disorder exposure to greater symptom severity (Griffiths et al. 2024), this co‐occurrence raises concerns about the safety of recovery‐hashtagged spaces as recovery‐supportive environments. Accordingly, engagement with recovery‐labeled material should not be assumed to be benign or therapeutically supportive, and clinicians might exercise caution when discussing or recommending engagement with TikTok recovery content. Some guidance has encouraged individuals in recovery to curate their social media by following recovery‐focused accounts and shaping their feeds to prioritize content perceived as supportive of recovery (e.g., Eating Disorders Victoria 2026). Our findings suggest that the first part of this guidance (following recovery‐focused accounts) will not be successful unless the user simultaneously enacts the second part of the guidance (active feed shaping). To assist clients to navigate these environments, clinicians might consider fostering social media literacy alongside recovery literacy, helping individuals critically evaluate online content, recognize disorder‐reinforcing elements embedded within recovery narratives, and implement strategies to minimize unwanted exposure via feed shaping (e.g., blocking pro‐eating disorder accounts). Clinicians could also educate clients on how to discuss recovery on social media without unwittingly reproducing pro‐eating disorder themes. These considerations may also be relevant for carers, families, and peer‐support networks who may assume recovery‐tagged content is inherently positive or supportive.

At the platform level, the findings underscore the importance of more context‐sensitive moderation and recommendation systems. Content such as calorie quantification or body‐focused comparison trends may be especially risky within recovery‐labeled spaces, even if tolerated elsewhere on the platform, because recovery hashtags likely function as help‐seeking or vulnerability signals, meaning users entering these spaces may be particularly susceptible to harmful or ambiguous content. Responsibility for managing these risks should not fall solely on individuals with eating disorders, their carers, or clinicians. Social media platforms and regulators also have a role in ensuring that recovery‐labeled spaces are as safe as reasonably possible, particularly for adolescents and younger users who may have reduced capacity to critically evaluate disorder‐reinforcing content. These findings also highlight the difficulty of moderating recovery‐labeled content, given that harmful messaging is often embedded within ostensibly supportive narratives rather than presented as overt pro‐eating disorder advocacy. Addressing these challenges will likely require both improved detection of disorder‐reinforcing rhetoric and more nuanced recommendation strategies that avoid collapsing recovery and pro‐eating disorder material within the same feed environments. Greater transparency around how recovery‐related content is ranked and clustered may also be warranted. In the absence of such safeguards, algorithmic systems may inadvertently amplify mixed or harmful content to users actively seeking recovery‐oriented material.

### 4.5 Future Directions for Research

Future research should examine how TikTok's recommendation systems shape exposure to recovery and pro‐eating disorder content over time. Experimental audit studies using simulated accounts could test whether engagement with recovery‐labeled content increases subsequent delivery of adjacent pro‐eating disorder material, and whether this differs across developmental groups such as adolescents versus adults. More broadly, further research is needed on how people with eating disorders navigate recovery‐oriented TikTok environments, particularly when recovery and pro‐eating disorder content are delivered alongside one another.

### 4.6 Study Limitations

Several limitations warrant consideration. First, reliance on hashtags to identify and categorize content may have resulted in misclassification or incomplete capture of relevant material, particularly given the use of coded, evasive, or absent tags. Second, although we attempted to minimize personalization by using incognito browsing and non‐logged‐in retrieval methods, some degree of algorithmic curation (e.g., country‐ or device‐level personalization) was likely unavoidable. Third, reflexive thematic analysis is inherently interpretive, and subtle in‐group language or symbolic cues may not have been fully captured. Fourth, eating disorder diagnoses were self‐reported and not confirmed via structured clinical interview, introducing potential diagnostic misclassification. Fifth, the quantitative sample was modest in size, limiting statistical power. Sixth, our results may not generalize beyond the populations most represented in our sample (i.e., young females with anorexia nervosa). Seventh, the cross‐sectional design precludes causal inference regarding the relationship between content exposure and symptom severity. Eighth and finally, we did not examine the temporal sequencing of pro‐eating disorder and recovery exposures within feeds; future research will explicitly investigate whether recovery exposure precedes, follows, or co‐evolves with disorder‐reinforcing content delivery.

## 5 Conclusion

This study demonstrates that eating disorder recovery content on TikTok exists within a digitally mediated ecosystem in which recovery‐aligned messaging, disorder‐reinforcing elements, and algorithmic co‐occurrence intersect. Recovery‐labeled posts are frequently contaminated with pro‐eating disorder features, and recovery content is typically delivered within feed environments that also contain substantial pro‐eating disorder material. Although we found no detectable protective association between recovery exposure and symptom severity in this sample, the entanglement of recovery and disorder‐reinforcing content suggests that TikTok recovery spaces cannot be assumed to be reliably safe or therapeutically supportive. Clinicians and platforms alike should approach these environments with caution and critical awareness.

## Author Contributions

**Scott Griffiths:** conceptualization, data curation, methodology, formal analysis, funding acquisition, project administration, resources, supervision, validation, visualization, writing – original draft, writing – review and editing. **Harish Kannan:** data curation, software, writing – review and editing, validation. **Emily Harris:** data curation, funding acquisition, investigation, methodology, project administration, software, validation, writing – review and editing. **Noa Brittain:** writing – review and editing. **Jasmine Fardouly:** writing – review and editing. **Isabel Martyn:** data curation, methodology, formal analysis, writing – original draft, writing – review and editing, visualization. **Emma Austen:** methodology, supervision, writing – review and editing. **Ben Stone:** data curation, software, validation, writing – review and editing. **Wesley Grey:** writing – review and editing, validation. **Deborah Mitchison:** writing – review and editing. **Edoardo Tescari:** data curation, software, writing – review and editing. **Usha Nattala:** data curation, software, writing – review and editing. **Jake Linardon:** writing – review and editing.

## Lived Experience Involvement Statement

No specific efforts were undertaken to involve persons with lived experience in the study design or execution, or in the preparation of this manuscript.

## Funding

Scott Griffiths receives funding from the National Health and Medical Research Council and the Medical Research Future Fund (grant numbers: 1179321, 1193738).

## Ethics Statement

All procedures were approved by the Human Research Ethics Committee at the University of Melbourne: ethics IDs #23072 and #32634. Informed consent was obtained from all participants.

## Conflicts of Interest

Scott Griffiths has provided unpaid expertise to Government bodies in Australia, Singapore, and the United Kingdom; unpaid expertise to Meta; and paid expertise to TikTok. The author affirms that no external parties or organizations had input into the design, conduct, analysis, or interpretation of this study or manuscript. Jake Linardon is an Associate Editor of the International Journal of Eating Disorders and co‐author of this article. Jake was excluded from editorial decision‐making related to the acceptance of this article for publication in the journal.

## Data Availability

Data are available at https://osf.io/tme25/overview.
